# The Rose Bengal Test in Human Brucellosis: A Neglected Test for the Diagnosis of a Neglected Disease

**DOI:** 10.1371/journal.pntd.0000950

**Published:** 2011-04-19

**Authors:** Ramón Díaz, Aurora Casanova, Javier Ariza, Ignacio Moriyón

**Affiliations:** 1 Departamento de Microbiología y Parasitología, Facultad de Medicina, Universidad de Navarra, Pamplona, Spain; 2 Departamento de Microbiología, Hospital Universitari de Bellvitge, Universidad de Barcelona (IDIBELL), Barcelona, Spain; 3 Departamento de Enfermedades Infecciosas, Hospital Universitari de Bellvitge, Universidad de Barcelona (IDIBELL), Barcelona, Spain; University of California San Diego School of Medicine, United States of America

## Abstract

Brucellosis is a highly contagious zoonosis affecting livestock and human beings. The human disease lacks pathognomonic symptoms and laboratory tests are essential for its diagnosis. However, most tests are difficult to implement in the areas and countries were brucellosis is endemic. Here, we compared the simple and cheap Rose Bengal Test (RBT) with serum agglutination, Coombs, competitive ELISA, Brucellacapt, lateral flow immunochromatography for IgM and IgG detection and immunoprecipitation with *Brucella* proteins. We tested 208 sera from patients with brucellosis proved by bacteriological isolation, 20 contacts with no brucellosis, and 1559 sera of persons with no recent contact or brucellosis symptoms. RBT was highly sensitive in acute and long evolution brucellosis cases and this related to its ability to detect IgM, IgG and IgA, to the absence of prozones, and to the agglutinating activity of blocking IgA at the pH of the test. RBT was also highly specific in the sera of persons with no contact with *Brucella*. No test in this study outperformed RBT, and none was fully satisfactory in distinguishing contacts from infected patients. When modified to test serum dilutions, a diagnostic titer >4 in RBT resulted in 87.4% sensitivity (infected patients) and 100% specificity (contacts). We discuss the limitations of serological tests in the diagnosis of human brucellosis, particularly in the more chronic forms, and conclude that simplicity and affordability of RBT make it close to the ideal test for small and understaffed hospitals and laboratories.

## Author Summary

The Rose Bengal Test (RBT) for brucellosis serological diagnosis was adapted to test serum dilutions and its usefulness evaluated using sera of *Brucella* culture positive patients, persons with contact with *Brucella* but no symptoms, veterinarians accidentally injected with vaccine Rev 1 who had not developed the disease and normal persons. Using the standard protocol, RBT was not outperformed by more sophisticated and expensive tests (serum agglutination, Coombs, competitive ELISA, Brucellacapt, and lateral flow immunochromatography for IgM and IgG detection) in identifying *Brucella* infected patients. All tests failed to discriminate with total specificity the sera from contacts or Rev 1 injected individuals. However, none of these sera was positive in the modified RBT adapted to test serum dilutions at titers higher than 1>4. When there is suspicion of brucellosis, RBT is recommended as the first test and, depending upon the titer, a positive result does not need confirmation by other (usually more expensive, sophisticated and time consuming) tests.

## Introduction

Brucellosis is a highly contagious zoonosis caused by the Gram-negative bacteria of the genus *Brucella*. *B. abortus*, *B. suis* and *B. melitensis*, three of the so-called smooth (S) brucellae, preferentially infect cattle, swine and sheep and goats, respectively. These animals are the source of most cases of human brucellosis, a grave and debilitating disease that may leave disabling sequelae. Its incidence is very high in some countries of the Mediterranean basin and bordering areas and, in all likelihood, in developing countries throughout the world. The reported incidence in these countries varies widely (from <0.01 to >200 per 100,000), reflecting the difficulties in recognizing a disease that lacks pathognomonic symptoms [1], [2]. This absence of specific symptoms makes it difficult to distinguish brucellosis from several febrile conditions that often occur in the same areas, including malaria [3]–[11] so that laboratory tests are essential for diagnosis [12]. Among these tests, only the isolation of the microorganism provides absolute proof of infection but bacteriological diagnosis is expensive and dangerous. On the other hand, serological tests are easier to implement and a great aid in diagnosis. The humoral immunoresponse to S brucellae is dominated by antibodies to the polysaccharide (PS) section of the *Brucella* S lipopolysaccharide (S-LPS) and it shows a typical IgM/IgG (and IgA) shift. In acute cases (i.e., short evolution) IgM is present in the serum; then this immunoglobulin returns progressively to background levels, so that IgG (and IgA) are dominant in the sera of long evolution (i.e. chronic) patients before treatment. Moreover, non-agglutinating antibodies (detected in the Coombs test) increase over agglutinating antibodies (active in the classical serum agglutination test [SAT]) during the course of the infection [12], [13]. Accordingly, the SAT-Coombs combination has been classically used both to increase sensitivity and to evaluate the stage of evolution of the infection. Other S-LPS (or PS) tests proposed more recently include the lateral flow immunochromatography assay (LFiC) for IgM and IgG assessment, a fluorescence polarization assay, a variety of indirect ELISA, and the immunocapture Brucellacapt test (for a recent review, see [14]). In addition, a competitive ELISA (cELISA) has been proposed [15]. Because these tests require well equipped laboratories and/or adequate budgets, they cannot be implemented in many laboratories in endemic areas.

The Rose Bengal test (RBT) is a rapid slide-type agglutination assay performed with a stained *B. abortus* suspension at pH 3.6–3.7 and plain serum. Because of its simplicity, it is often used as a screening test in human brucellosis and would be optimal for small laboratories with limited means. However, there is confusion about the value of this test so that present WHO guidelines recommend that RBT results be confirmed by other tests [14], [16]. Points of concern expressed by several authors include low sensitivity [17] particularly in long evolution (chronic) cases [18], [19] and relatively low specificity in endemic areas [20], [21]. Moreover, some authors consider that prozones make strongly positive sera appear as negative in RBT [22]. In the present work, we have addressed these points and reexamined the usefulness of RBT for the diagnosis of human brucellosis using sera of patients with no brucellosis, culture-positive brucellosis patients, and healthy persons that had had contact with the pathogen.

## Materials and Methods

### Ethical statement

The sera used in this work were obtained during clinical practice in the 1975–2001 period. Their use in this research was approved by the Ethical Boards of Clínica Universidad de Navarra (Pamplona, Spain), Hospital de Navarra (Pamplona, Spain), Hospital Universitari de Bellvitge (Barcelona, Spain), Hospital Clínico Universitario Virgen de la Victoria (Málaga, Spain), Hospital de la Inmaculada de Huércal-Overa (Almería, Spain), Hospital General Universitario de Albacete (Albacete, Spain), and Hospital Clínico Universitario de Valladolid (Valladolid, Spain).

### Serological tests

For RBT, 30 µL of plain serum were dispensed on a white glossy ceramic tile and mixed with an equal volume of RBT antigen (Veterinary Laboratory Agency; England, United Kingdom; http://www.defra.gov.uk/vla/) (previously equilibrated at room temperature and shaken to resuspend any bacterial sediment) using a toothpick. The tile was then rocked at room temperature for 8 minutes (instead of the 4 minutes recommended for animal brucellosis [23]), and any visible agglutination and/or the appearance of a typical rim [23] (Figure S1) was taken as a positive result. Positive sera were tested further as follows. Eight 30 µL drops of saline were dispensed on the tile and the first one mixed with an equal volume of the positive plain serum (1/2 serum dilution). Then, 30 µL of this first dilution were transferred to the second drop with the help of a micropipette and mixed to obtain the 1/4 dilution. From this, the 1/8 to 1/128 dilutions were obtained by successive transfers and mixings taking care of rinsing the pipette tip between transfers. Finally, each drop was tested with an equal volume (30 µL) of the RBT reagent, so that the final dilutions ranged from 1/4 to 1/256. The SAT and Coombs test in microtitter plates, Brucellacapt (Vircell S.L, Santa Fe, Granada, Spain) and LFiC (kindly provided by Dr. H. Smits, KIT Biomedical Research, Royal Tropical Institute/Koninklijk Instituut voor de Tropen, Amsterdam, The Netherlands) were performed as described before [24], [25]. For the Coombs test, a titer ≥ two times the SAT titer in the same serum was considered as positive. In some cases (see Results), SAT was also performed in the citrate buffer (pH 5) provided as diluent in the Brucellacapt kit. To this end, the bacteria in a volume of the SAT suspension were collected by centrifugation, washed with citrate buffer and resuspended in an equal volume of the same buffer. cELISA was performed according to the instructions of the manufacturer (Svanova Biotech, Uppsala, Sweden). Antibodies to *Brucella* proteins were detected by counterimmunoelectrophoresis (CIEP) using an S-LPS free extract obtained from a *B. melitensis* rough mutant [26].

### Human sera

The following groups of sera were used: (i), two hundred and eight sera of an equal number of patients with brucellosis confirmed by bacteriological culture (all *B. melitensis*) that were diagnosed at the above-mentioned institutions in the 1975–2001 period; a subset of patients in this group for which the IgM and IgG profile could be determined (by LFiC) were classified as short (IgM dominant) or longer evolution (IgG dominant with low or no IgM) (see Results) and correspond broadly to the concepts of acute and chronic brucellosis; (ii), the sera of 20 persons (Table 1 in Supporting Information S1) that had had professional contact (veterinarians, slaughter house workers, shepherds, etc.) with *B. melitensis*-infected animals or their products or had accidentally injected themselves with vaccine *B. melitensis* Rev 1 and that were followed for a period of at least two years; (iii), eleven sera from brucellosis patients that had been collected in a different study because they showed the prozone effect; and (iv), one thousand five hundred and fifty-nine sera from patients with no symptoms of brucellosis sent to the laboratory for the serological diagnosis of other infections. The sera were aliquoted and kept frozen at −20°C. Care was taken not to thaw and freeze repeatedly these sera. Controls showed no deterioration under these conditions.

**Table 1:** Results of SAT and RBT in patients (n = 208) with brucellosis proved by bacteriological culture.^1^

SAT		N° of positive sera using the standard RBT protocol (%)	N° of RBT positive (%) at titers:^2^	
Titer	N° of sera		≤4	≥8
≤1∶20	6	6 (100)	2 (0,96)	4 (1,92)
≥1∶40	202	202 (100)	26 (12,5)	176 (85,5)
≥1∶80	201	201 (100)	25 (12,0)	176 (85,0)
≥1∶60	185	185 (100)	13 (6,20)	172 (83,0)
≥1∶320	160	160 (100)	2 (0,90)	158 (76,3)
≥1∶640	136	136 (100)	0 (0,00)	136 (65,7)

1 Sera were collected from patients with brucellosis proved by blood (n = 205) or abscess (n = 3) culture.

2 Titers correspond to plain serum (titer 1∶2) or serum dilutions made in phosphate buffered saline and then tested with an equal volume of RBT regent (1∶4, etc.).

## Results

The 1559 sera from patients with no brucellosis yielded only one positive result in the standard RBT. The patient was asymptomatic and re-examination of the medical history showed that he had suffered from brucellosis in the past. The sera of 19 of the 20 persons that had had professional contact with *B. melitensis*-infected animals or had accidentally injected themselves with vaccine Rev 1 showed reactions in the standard RBT despite the fact that these persons were consistently asymptomatic. None of these sera, however, had a titer >1∶4 when tested in the modified RBT (Table 1 in Supporting Information S1). In one case (C-20, Table 1 in Supporting Information S1), seroconversion was observed at the time when symptoms compatible with brucellosis developed, and this patient was successfully treated with antibiotics. Concerning other tests, 3 of these 20 persons had SAT titers equal to 160, 8 had Brucellacapt titers ≥320, 16 had a positive Coombs, 4 and 8 were LFiC-IgM and -IgG positive, respectively, and 5 showed antibodies to cytosolic proteins.

Table 1 compares the results of SAT and RBT obtained with the sera of the 208 culture positive patients. Whereas 185 had SAT titers ≥160, the standard RBT identified as positive all the 208 sera. When performed on serum dilutions, a RBT titer discriminating all healthy contacts (≥1∶8; previous paragraph) would identify correctly 180 sera of the culture positive patients (176+4; Table 1; 87.4% sensitivity). A SAT titer similarly discriminating all healthy contacts (>1∶160; previous paragraph) would identify only160 of these patients (76.9% sensitivity). In those cases that could be studied with more detail, RBT titers varied from 4 to 256 in the sera with weak or negative Coombs and anti-S-LPS IgM but no IgG, and from 4 to 128 in the sera with a positive Coombs and anti-S-LPS IgG stronger than IgM (Tables 2 and 3 in Supporting Information S1).

**Table 2:** Results of serological tests with sera showing the SAT blocking phenomenon.^1^

	Reciprocal of serum titers in:								
		SAT					LFiC^2^		CIEP-proteins^3^
Patient N°	RBT	pH 7^4^	pH 7, IgA- absorbed^5^	pH 5^6^	Brucellacapt	Coombs-IgG	IgM	IgG	
1.	4	<20	40	160	640	1280	0	2	4 (3)
2.	16	<20	160	640	5120	10240	0	3	64 (4)
3.	16	<20	40	640	5120	20480	0	4	32 (5)
4.	2	<20	20	160	640	2560	0	3	16 (3)
5.	32	<20	ND^7^	1280	10240	40960	0	4	64 (8)
6.	8	<20	320	ND^7^	ND^7^	20480	0	ND^7^	8 (3)

1 Sera were collected from patients with brucellosis proved by blood (n = 5) or abscess (n = 1) culture.

2 From 0 (negative) to 4 (strong positive).

3 Reciprocal of serum titers (number of precipitin lines).

4 Standard SAT with antigen and serum dilutions in PBS pH 7.

5 SAT performed in PBS pH 7.0 using serum deprived of IgA by absorption with anti-human IgA rabbit serum.

6 SAT performed with antigen resuspended in citrate pH 5 and serum dilutions in the same buffer.

7 ND, not done.

**Table 3:** Results with sera showing SAT titers <1∶160 and no blocking phenomenon.^1^

Patient N°	Serum titers:					LFiC^2^		
	RBT	SAT	Brucellacapt	Coombs-IgG	cELISA %^3^	IgM	IgG	CIEP-proteins^4^
7.	4	80	ND	80	ND^5^	2	ND^5^	0
8.	2	40	640	320	35	2	0	1 (1)
9.	4	80	640	640	42	2	0	4 (2)
10.	2	80	640	320	45	2	1	1 (1)
11.	4	80	640	640	35	1	2	8 (3)
12.	4	80	640	1280	51	1	2	4 (2)
13.	4	80	640	1280	53	1	2	8 (1)
14.	4	40	40960	20480	95	+/−	3	2 (1)
15.	2	40	320	160	55	0	0	0
16.	4	80	1280	640	32	0	0	8 (1)
17.	2	40	320	2560	55	0	0	0
18.	4	80	640	1280	38	0	1	0
19.	2	80	640	1280	39	0	2	16 (4)
20.	2	40	5120	10240	ND^5^	0	3	16 (4)
21.	4	80	2560	10240	75	0	3	32 (4)
22.	4	80	ND	320	ND^5^	ND^5^	ND^5^	1 (1)
23.	2	80	ND	2560	ND ND	ND^5^	ND^5^	16 (3)

1 Sera were collected from patients with brucellosis proved by blood (n = 15) or abscess (n = 2) culture.

2 From 0 (negative) to 4 (strong positive).

3 % competitive index.

4 Serum titers (number of precipitin lines).

5 ND, not done.

The 23 sera (208 -185) of culture positive patients with SAT titers <160 included 6 showing the blocking phenomenon. This is a rare event appearing in some prolonged brucellosis cases when non-agglutinating IgA are in amounts higher than other anti-S-LPS antibodies and it represents the extreme case of prozones [27]–[30]. Table 2 shows that neither RBT nor Brucellacapt were affected by the blocking phenomenon and that, as expected, SAT titers increased upon IgA removal. Since RBT and Brucellacapt have in common the use of acid buffers (pH 3.65 and 5.0, respectively), we hypothesized that an acid pH could promote agglutination and overcome the presence of blocking IgA. To test this, we substituted citrate buffer pH 5.0 for the saline in the standard SAT bacterial suspension (see Material and Methods). Under these conditions, the blocking activity in SAT disappeared. To confirm that the use of an acid buffer removes the agglutination-inhibitory effect of IgA and accounts for the absence of prozones in RBT, we examined 11 sera showing 1/40 to 1/80 prozones. These prozones disappeared upon absorption with anti-IgA and were not observed in SAT at pH 5, RBT or Brucellacapt.

Of the 23 sera with SAT titers <160, 2 (n°s 1 and 4, Table 2) and 17 (n°s 7 to 23, Table 3) had RBT titers ≤4. Using these 23 sera and those of the 20 persons that had had professional contact with *B. melitensis*-infected animals or had accidentally injected themselves with vaccine Rev 1 (see above), we examined whether other tests could complement RBT and discriminate the sera of infected patients from those of healthy contacts (Table 1 in Supporting Information S1). The numbers of false positives/false negatives were: CIEP-proteins, 5/4; LFiC-IgM, 4/13; LFiC-IgG, 12/5; Brucellacapt (≥1∶640), 2/2; and Coombs (≥two times the corresponding SAT titer) 18/1, and cELISA (cut-off at 30% inhibition) 8/0.

Finally, 11 culture positive patients could be followed periodically. SAT, LFiC-IgM, LFiC-IgG and RBT became negative between months 1 and 16 after the beginning of a successful antibiotic treatment. However, 4 and 2 of these patients remained positive in Coombs, and cELISA, respectively, and 2 had Brucellacapt titers equal to 1∶320. Although greatly diminished in titer and number of precipitin lines, 6 patients remained positive in CIEP (a single precipitin line in all cases).

## Discussion

One of the early findings in brucellosis was the observation that the sera of infected individuals contained agglutinating antibodies that could be detected in SAT. This test was soon adapted to the more practical slide-agglutination format but this method was prone to false negative results because of prozones and blocking and non agglutinating antibodies [31]. We show here that RBT overcomes these three problems. Moreover, we confirm that it is highly sensitive and demonstrate that a simple adaptation to test serum dilutions improves its specificity and considerably reduces the need for additional serological tests. This simple modification makes RBT close to the ideal test for small laboratories.

Although the overall sensitivity reported for RBT varies widely, there could be several reasons for this. Variations in sensitivity have been demonstrated in the past for RBT antigens of various sources [32], [33] and the use of good quality antigens made by experienced or reference laboratories is of the utmost importance. Although this has been occasionally considered as a weakness of RBT [14], it is well know that a good quality control is necessary in all brucellosis serological tests because of the tendency of S brucellae to dissociate into rough variants lacking the diagnostically significant S-LPS epitopes [23]. Also, the use of white opaque glossy surfaces is important [34], and awareness of the various agglutination patterns (Figure S1a proper incubation time is critical. With regard to the latter, the literature shows from 2 to 5 minutes [20], [34], [35]. However, it has been known for a long time [36] that some human sera require a longer incubation to become positive in the RBT-like card test. In our experience, sera without blocking antibodies or prozones are strongly positive in less than 4 minutes, but sera with blocking IgA or with high titers of non-agglutinating antibodies (high Coombs titers) may need up to 8 minutes to develop the bacterial clumps or the characteristic rim. These antibodies are typical of long evolution brucellosis and, therefore, the low sensitivity (54 to 61%) reported in chronic brucellosis by some authors [18], [19] could be accounted for by a non optimized RBT protocol. Our results clearly show that RBT was equally useful in the IgM-negative (longer evolution) and IgM-positive (shorter evolution) groups of patients and that the use of an acidic pH abrogates prozones and blocking phenomena.

Consistent with the demonstration that RBT detects both S-LPS specific IgM, IgG and IgA and that neither prozones nor blocking antibodies are sources of false negative results, most authors have reported a high sensitivity in culture-positive patients, equal or better than that of SAT, ELISA-IgG, ELISA-IgM, or LFiC for IgM plus IgG [20], [37]–[39]. In this work, we have also used the sera of culture positive patients as the reference, and this point deserves attention for a correct understanding of our results in a clinical context. Careful studies with appropriate bacteriological procedures have shown that the rate of success in isolating *Brucella* is higher during the initial disease than in relapses (c.a. 80 versus 65%, respectively, in ref. [40]) and lowest in the more chronic forms [41]. The reasons for this consistent observation are not understood but, as illustrated for the case of hepatosplenic abscesses [25], [41], it is in a fraction of the more chronic cases where serology (by RBT or other tests) and culture are sometimes not conclusive. Indeed, the scarce RBT negative results that have been well documented correspond to a few patients with focal forms of brucellosis [25], [41]–[43]. In these difficult cases, a combination of serological tests and clinical findings and a careful follow-up of the patients are in order. The evidence obtained in a limited number of these cases suggests that the Coombs test provides the best indication of the seroconversion that parallels the relapses and the evolution during treatment [25], [44] (see also below).

The specificity of the RBT and other S-LPS tests is also worth commenting on. Febrile conditions including tuberculosis, malaria, typhoid fever, Still's disease, lupus erythematosus, rheumatoid arthritis, sarcoidosis, and active lymphoma are not a source of RBT false positive results [5]. On the other hand, S-LPS cross-reactivity with *Vibrio cholerae*, *Francisella tularensis* and *Yersinia enterocolitica* 0:9 is a potential source of unspecific results in all S-LPS tests. However, this is of little importance in clinical practice. Although positive cases have been reported in *V. cholerae* vaccinated individuals [45], [46], there are no RBT observations in cholera patients and this illustrates that the clinical picture is widely different. With regard to tularemia, in a series of 5 patients, 3 were RBT positive (T. Marrodán, Ph. Thesis, University of Navarra, Spain) but these were easily differentiated by the clinical picture and other tests. Yersiniosis by *Y. enterocolitica* O:9 elicits antibodies that react in all *Brucella* S-LPS tests including RBT [26], [47] but there are tests with protein antigens that discriminate *Y. enterocolitica* O:9 and S *Brucella* infections [26] (see also below). Indeed, antibodies to *Brucella* S-LPS persist for protracted periods in a percentage of recovered patients in all S-LPS tests [13]. Therefore, a past history of brucellosis is a cause of unspecific serological results that has to be evaluated by the physician. Finally, some authors consider that RBT has a limited usefulness in endemic areas [20], [21]. However, Ruiz-Mesa et al. [48] compared the sera of individuals that had had repeated contact with *Brucella* with those with no regular exposure or history of brucellosis, and reported specificities of 91.7 and 94.3%. This same problem was addressed by Gómez et al. [39] who found 100 and 97% sensitivity and specificity, respectively. These studies indicate that the specificity problem of the standard RBT is not so critical and that, as illustrated by our results, other S-LPS tests are also affected. In this regard, it is important to stress that the diagnosis of human brucellosis has to be made on the basis of compatible symptoms, clinical findings and a thorough anamnesis, that it cannot rely exclusively on a weak positive result in any S-LPS serological test and that there are no cut-off diagnostic titers in any single S-LPS test. An alternative to S-LPS tests is the use of protein tests [12]. It has been known for a long time that a large proportion of brucellosis patients develop antibodies to soluble *Brucella* proteins [26]. However, in the present study, 5 of the 20 healthy persons that had had professional contact with infected livestock developed anti-protein antibodies (Table 1 in Supporting Information S1). Recently, a large number of *Brucella* proteins have been evaluated by Liang et al. [49] using brucellosis patients that were culture and RBT-positive and had SAT titers ≥1∶160 as well as healthy persons. For a combination of the 5 top serodiagnostic proteins, these authors reported a specificity of 96% (95% sensitivity, both values optimized by ROC analysis). Although further studies are necessary to reach a definite conclusion, these data suggest that protein antigens may not completely solve the specificity problems in human brucellosis serodiagnosis.

**Figure 1:**
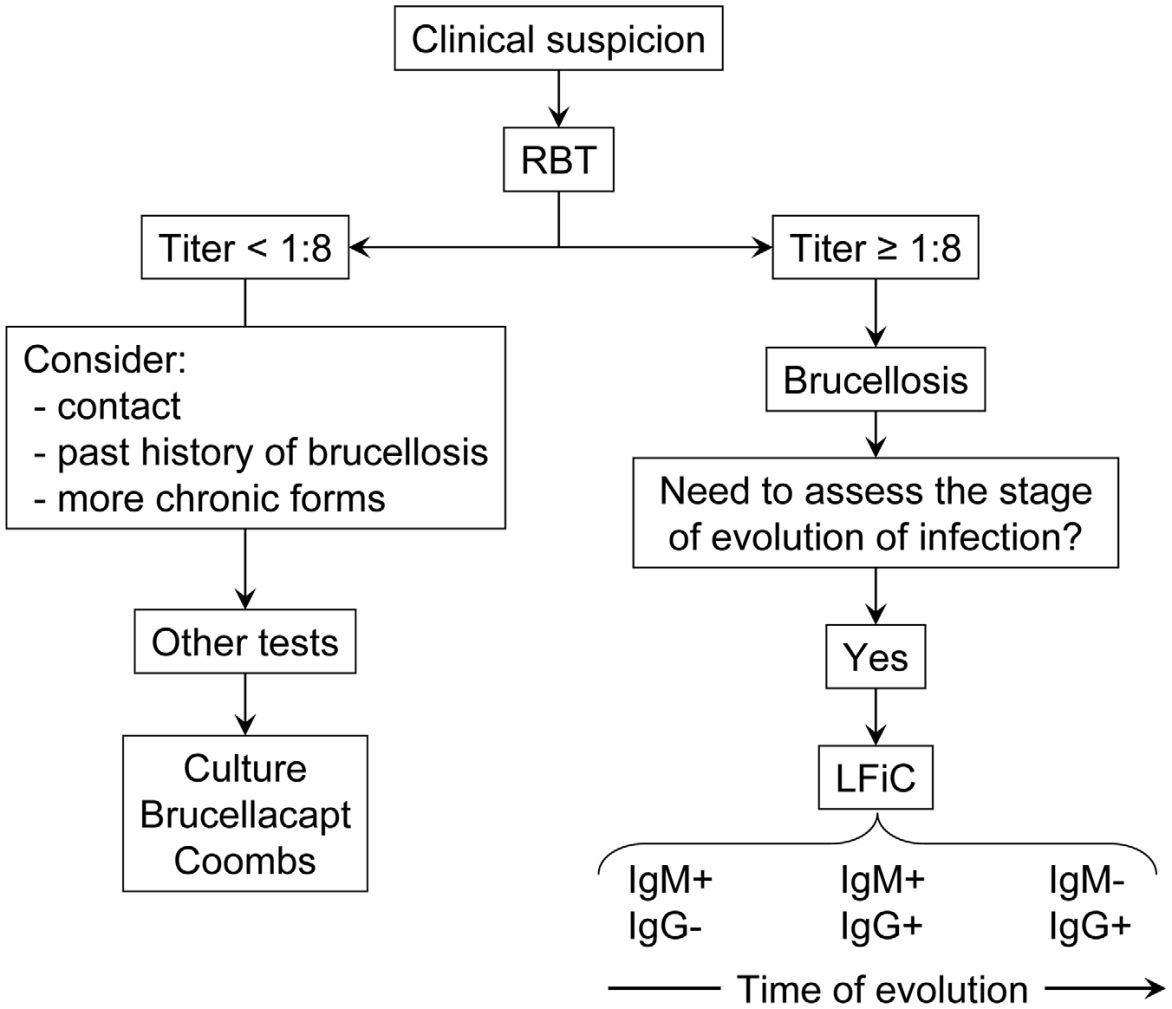
Proposed use of RBT in the diagnosis of human brucellosis and complementary tests.

In summary, when complemented with appropriate anamnesis and clinical findings, RBT is a very useful test for the diagnosis of human brucellosis. It needs no complicated infrastructure or sophisticated training, it is exceedingly cheap, highly sensitive and easily adaptable to test serum dilutions. On these bases, a simple scheme for the diagnosis of human brucellosis can be proposed that could avoid confirmation of a large proportion of positive results in the standard RBT protocol (i.e., those RBT titers ≥1∶8) (Figure 1). If an assessment of the stage of evolution of a particular case is necessary, a complementary test assessing IgM and IgG levels could be used, and for this purpose the simple LFiC seems the appropriate complement to RBT in small laboratories. Indeed, it is not possible to predict the proportion of RBT results that need confirmation (RBT titers <1∶8; Figure 1) in a given population but it is expected that long evolution cases with low levels of antibodies in RBT or other S-LPS tests would be more common in endemic areas with no adequate awareness of the human disease. As stressed above, these cases would need a careful assessment by physicians, and further serological studies in well equipped laboratories using tests like Coombs or Brucellacapt. Moreover, culture should be attempted because, even though its sensitivity is low in these cases, a positive result is a definite proof of infection.

## Supporting Information

Figure S1 Degrees of agglutination in RBT.
(0.17 MB DOC)

Supporting Information S1 Results with sera of contacts and serologically defined shorter and longer evolution cases.
(0.27 MB DOC)

Checklist S1 STARD Table Checklist.
(0.05 MB DOC)
