# Supplementary figures and images for: The Rose Bengal Test in Human Brucellosis: A Neglected Test for the Diagnosis of a Neglected Disease
Source: PLoS Negl Trop Dis. 2011 Apr 19;5(4):e950. doi: 10.1371/journal.pntd.0000950 (PMC3079581; doi:10.1371/journal.pntd.0000950)

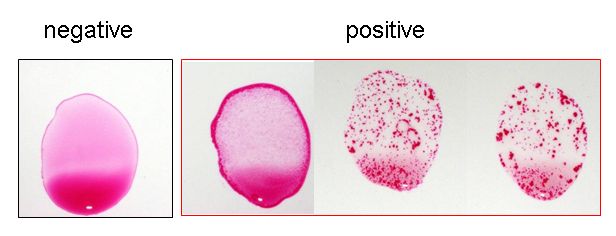


Figure S1. Different degrees of agglutination in RBT

Supplement: Figure S1 — Degrees of agglutination in RBT. (0.17 MB DOC) [file pntd.0000950.s001.doc]
