# Supplementary material for: The Rose Bengal Test in Human Brucellosis: A Neglected Test for the Diagnosis of a Neglected Disease
Source: PLoS Negl Trop Dis. 2011 Apr 19;5(4):e950. doi: 10.1371/journal.pntd.0000950 (PMC3079581; doi:10.1371/journal.pntd.0000950)
Supplement: Supporting Information S1 — Results with sera of contacts and serologically defined shorter and longer evolution cases. (0.27 MB DOC) [file pntd.0000950.s002.doc]

Supporting Information S1 (Tables)

Table 1. Serological results in persons that had professional contact with *Brucella* but no symptoms.

|  | S-LPS tests: | | | | | | |  |
| --- | --- | --- | --- | --- | --- | --- | --- | --- |
|  | Serum titers: | | | |  | LFiC ^3^ | |  |
| Group / Code ^1^ | RBT | SAT | Brucapt | Coombs IgG | cELISA % ^2^ | IgM | IgG | CIEP-proteins ^4^ |
| Abattoir worker |  |  |  |  |  |  |  |  |
| C-1 (0) | 4 | 40 | 320 | 5120 | 65 | 0 | 3 | 1(1) |
| C-1 (24) | 4 | 40 | 320 | 5120 | 52 | 0 | 3 | 1(1) |
| C-2 | 2± | 0 | 0 | 0 | -14 | 0 | 0 | 0 |
| C-3 | 2± | 40 | 40 | 160 | 31 | 0 | 0 | 0 |
| Meat processing plant worker |  |  |  |  |  |  |  |  |
| C-4 | 2 | 40 | 320 | 2560 | 48 | 0 | 2 | 0 |
| C-5 | 2 | 160 | 80 | 640 | 25 | 0 | 2 | 0 |
| Veterinarians accidentally injected with vaccine Rev1 |  |  |  |  |  |  |  |  |
| C-6 (0) | 4 | 40 | 2560 | 5120 | 80 | 0 | 3 | 1(1) |
| C-6 (18) | 4 | 40 | 640 | 1280 | 83 | 0 | 2 | 1(1) |
| C-7 | 2 | 0 | 320 | 2560 | 45 | 2 | 0 | 0 |
| C-8 | 2 | 80 | 320 | 1280 | 25 | 2 | 0 | 1(1)1 |
| C-9 | 4 | 80 | 80 | 1280 | 10 | 1 | 1 | 0 |
| C-10 | 2 | 80 | 80 | 160 | 20 | 1 | 1 | 0 |
| Veterinarians that had contacts with infected animals |  |  |  |  |  |  |  |  |
| C-11 | 2 | 80 | 160 | 1280 | 17 | 0 | 1 | 0 |
| C-12 | 2 | 40 | 80 | 320 | 3 | 0 | 0 | 0 |
| C-13 | 2± | 40 | 40 | 40 | 19 | 0 | 0 | 0 |
| C-14 | 2± | 20 | 20 | 160 | 3 | 0 | 0 | 0 |
| C-15 | 2± | 80 | 80 | 640 | 20 | 0 | 1 | 0 |
| C-16 | 2± | 40 | 80 | 640 | 24 | 0 | 0 |  |
| C-17 | 2 | 0 | 320 | 2560 | 22 | 0 | 2 | 0 |
| C-18 | 4 | 160 | 320 | 640 | 50 | 0 | 0 | 0 |
| C-19 | 4 | 160 | 320 | 2560 | 24 | 0 | 1 | 0 |
| C-20 (0) | 0 | <20 | <20 | 20 | 3 | ND^5^ | ND^5^ | 0 |
| C-20 (6) | 8 | 640 | ND^5^ | 2560 | ND^5^ | ND^5^ | ND^5^ | 4 (3) |

^1^ For those persons that were sampled repeatedly, figures in parenthesis indicate the months at which the samples were taken

^2^ % competitive index.

3 From 0 (negative) to 4 (strong positive).

^4^ Serum titers (number of precipitin lines).

^5^ ND, not done.

Table 2. Results of serological tests in culture positive patients (n = 21) with acute brucellosis.^1^

|  | Serum titers: | | | LFiC ^2^ | |  |
| --- | --- | --- | --- | --- | --- | --- |
| Patient Nº | RBT | SAT | Coombs-IgG | IgM | IgG | CIEP-proteins ^3^ |
|  | 4 | 160 | 640 | 2 | 0 | 0 (0) |
|  | 16 | 640 | 1280 | 4 | 0 | 0 (0) |
|  | 16 | 640 | 640 | 3 | 0 | 0 |
|  | 8 | 640 | 1280 | 3 | 0 | 0 (0) |
|  | 16 | 640 | 1280 | 4 | 0 | 0 (0) |
|  | 64 | 640 | 640 | 3 | 0 | 0 (0) |
|  | 16 | 640 | 1280 | 3 | 0 | 0 (0) |
|  | 8 | 1280 | 2560 | 2 | 0 | 8 (2) |
|  | 8 | 1280 | 2560 | 3 | 0 | 8 (2) |
|  | 73 | 1280 | 1280 | 3 | 0 | 0 |
|  | 16 | 1280 | 2560 | 3 | 0 | 0 (0) |
|  | 16 | 1280 | 1280 | 3 | 0 | 0 (0) |
|  | 16 | 2560 | 2560 | 4 | 0 | 1 (1) |
|  | 32 | 2560 | 2560 | 4 | 0 | 2 (1) |
|  | 32 | 2560 | 5120 | 3 | 0 | 0 (0) |
|  | 16 | 2560 | 2560 | 4 | 0 | 8 (2) |
|  | 32 | 2560 | 10240 | 4 | 0 | 16 (3) |
|  | 32 | 5120 | 5120 | 4 | 0 | 4 (1) |
|  | 128 | 5120 | 5120 | 4 | 0 | 16 (2) |
|  | 256 | 10240 | 20480 | 4 | 0 | 8 (2) |
|  | 128 | 10240 | 10240 | 4 | 0 | 16 (3) |

^1^ Defined serologically (IgM but no IgG by LFiC).

^2^ From 0 (negative) to 4 (strong positive).

^3^ Serum titers (number of precipitin lines).

^4^ n.d., no datum

Table 3. Results of serological tests in culture positive patients (n = 16) with long evolution brucellosis.^1^

|  | Serum titers: | | |  | LFiC ^2^ | |  |
| --- | --- | --- | --- | --- | --- | --- | --- |
| Patient Nº | RBT | SAT | Coombs-IgG |  | IgM | IgG | CIEP-proteins ^3^ |
| 2 ^4^ | 16 | <20 | 10240 |  | 0 | 3 | 64 (4) |
| 3 ^4^ | 16 | <20 | 20480 |  | 0 | 4 | 32 (5) |
|  | 8 | 160 | 40960 |  | 0 | 3 | 8 (2) |
|  | 4 | 160 | 1280 |  | 0 | 3 | 16 (5) |
|  | 4 | 320 | 2560 |  | 0 | 2 | 16 (2) |
|  | 8 | 320 | 20480 |  | 0 | 4 | 8 (3) |
|  | 16 | 320 | 1280 |  | 0 | 3 | 32 (2) |
|  | 16 | 320 | 10240 |  | 0 | 3 | 16 (3) |
|  | 16 | 640 | 5120 |  | 1 | 4 | 16 (2) |
|  | 32 | 1280 | 40960 |  | 0 ^5^ | 4 | 128 (6) |
|  | 16 | 1280 | 10240 |  | 0 | 3 | 32 (2) |
|  | 32 | 1280 | 10240 |  | 0 | 3 | 16 (3) |
|  | 64 | 2560 | 40960 |  | 1 | 4 | 16 (3) |
|  | 128 | 5120 | 40960 |  | 1 | 4 | 128 (3) |
|  | 64 | 1280 | 40960 |  | 0 | 4 | 16 (4) |
|  | 32 | 1280 | 20480 |  | 0 ^5^ | 3 | 128 (4) |

^1^ Defined serologically (IgM < IgG by LFiC).

^2^ From 0 (negative) to 4 (strong positive).

^3^ Serum titers (number of precipitin lines).

^4^ Patients with blocking antibodies (Table 3).

^5^ Sera giving false positive results in LFiC-IgM (negative upon absorption with anti-rheumatoid factor antibodies).
